# Adenosine Conjugated Docetaxel Nanoparticles—Proof of Concept Studies for Non-Small Cell Lung Cancer

**DOI:** 10.3390/ph15050544

**Published:** 2022-04-28

**Authors:** Hibah M. Aldawsari, Sima Singh, Nabil A. Alhakamy, Rana B. Bakhaidar, Abdulrahman A. Halwani, Nagaraja Sreeharsha, Shaimaa M. Badr-Eldin

**Affiliations:** 1 Department of Pharmaceutics, Faculty of Pharmacy, King Abdulaziz University, Jeddah 21589, Saudi Arabia; nalhakamy@kau.edu.sa (N.A.A.); rbakhaidar@kau.edu.sa (R.B.B.); aahalwani@kau.edu.sa (A.A.H.); smbali@kau.edu.sa (S.M.B.-E.); 2 Center of Excellence for Drug Research and Pharmaceutical Industries, King Abdulaziz University, Jeddah 21589, Saudi Arabia; 3 IES Institute of Pharmacy, IES University Campus, Kalkheda, Ratibad Main Road, Bhopal 462044, India; simasingh87@gmail.com; 4 Department of Pharmaceutical Sciences, College of Clinical Pharmacy, King Faisal University, Al-Ahsa 31982, Saudi Arabia; 5 Department of Pharmaceutics, Vidya Siri College of Pharmacy, Off Sarjapura Road, Bangalore 560035, India

**Keywords:** docetaxel, adenosine receptors, PLGA, nanoparticles, lung cancer

## Abstract

Non-small cell lung cancer, a molecularly diverse disease, is the most prevalent cause of cancer mortality globally. Increasing understanding of the clinicopathology of the disease and mechanisms of tumor progression has facilitated early detection and multimodal care. Despite the advancements, survival rates are extremely low due to non-targeted therapeutics and correspondingly increased risk of metastasis. At some phases of cancer, patients need to face the ghost of chemotherapy. It is a difficult decision near the end of life. Such treatments have the capability to prolong survival or reduce symptoms, but can cause serious adverse effects, affecting quality of life of the patient. It is evident that many patients do not die from burden of the disease alone, but they die due to the toxic effect of treatment. Thus, increasing the efficacy is one aspect and decreasing the toxicity is another critical aspect of cancer formulation design. Through our current research, we tried to uncover both mentioned potentials of the formulation. Therefore, we designed actively targeted nanoparticles for improved therapeutics considering the overexpression of adenosine (ADN) receptors on non-small cell lung cancer (NSCLC) cells. Docetaxel (DTX), an essential therapeutic as part of combination therapy or as monotherapy for the treatment of NSCLC, was encapsulated in biodegradable poly(lactic-co-glycolic acid) nanoparticles. ADN was conjugated on the surface of nanoparticles using EDC-NHS chemistry. The particles were characterized in vitro for physicochemical properties, cellular uptake, and biocompatibility. The size and zeta potential of DTX nanoparticles (DPLGA) were found to be 138.4 ± 5.45 nm and −16.7 ± 2.3 mV which were found to change after ADN conjugation. The size was increased to 158.2 ± 6.3 nm, whereas zeta potential was decreased to −11.7 ± 1.4 mV for ADN-conjugated DTX nanoparticles (ADN-DPLGA) indicative of surface conjugation. As observed from transmission electron microscopy (TEM), the nanoparticles were spherical and showed no significant change in encapsulation efficiency even after surface conjugation. Careful and systematic optimization leads to ADN-conjugated PLGA nanoparticles having distinctive characteristic features such as particle size, surface potential, encapsulation efficacy, etc., that may play crucial roles in the fate of nanoparticles (NPs). Consequently, higher cellular uptake in the A549 lung cancer cell line was exhibited by ADN-DPLGA compared to DPLGA, illustrating the role of ADN receptors (ARs) in facilitating the uptake of NPs. Further in vivo pharmacokinetics and tissue distribution experiments revealed prolonged circulation in plasma and significantly higher lung tissue distribution than in other organs, dictating the targeting potential of the developed formulation over naïve drug and unconjugated formulations. Further, in vivo acute toxicity was examined using multiple parameters for non-toxic attributes of the developed formulation compared to other non-targeted organs. Further, it also supports the selection of biocompatible polymers in the formulation. The current study presents a proof-of-concept for a multipronged formulation technology strategy that might be used to maximize anticancer therapeutic responses in the lungs in the treatment of NSCLC. An improved therapeutic and safety profile would help achieve maximum efficacy at a reduced dose that would eventually help reduce the toxicity.

## 1. Introduction

Lung cancer is the greatest cause of death and illness in the world (1.59 million deaths per year), followed by colon and liver cancer. NSCLC (non-small cell lung cancer) constitutes roughly 85% of all bronchogenic carcinomas [1] that pose a relentless threat to human health [2]. It is marked by a high proliferative rate, a strong predilection for metastasis, and a poor prognosis. More than 70% of NSCLC patients are elderly, current, or past heavy smokers, and the risk rises with increasing duration and intensity of smoking [3]. Although the disease is highly sensitive to chemotherapy and radiation, a higher dose of radiation causes severe damage to normal tissues around the tumor, causing poor patient compliance and therapeutic outcome [4]. On the other side, conventional chemotherapy has its shortcomings, such as nonspecific biodistribution, toxicity, etc. [5,6].

Cancer nanotherapeutics are rapidly evolving to solve several limitations of conventional drug delivery systems [7]. The ideal physicochemical characteristics that jointly confer molecular targeting, immune evasion, and controlled drug release have been a fundamental barrier to effective clinical translation of anticancer nanomedicines. Increasing understanding of the clinicopathology of the disease and mechanisms of tumor progression has proved that adenosine (ADN) receptors (ARs) are over-expressed on tumor cells of NSCLC [8]. There are multiple subtypes of ARs that are being explored, i.e., A1, A2A, A2B, and A3, for cancer research [9], though primarily A3ARs were found to be upregulated in multiple cancers including NSCLS [10]. This is the reason why the A3AR was considered to be the tumor marker. Extracellular ADN induces apoptosis in cancer cells via diverse signaling pathways linked to ARs. ADN and other AR agonists might be effective in preventing or slowing the progression of NSCLC and other cancers [11]. Although there are many studies on the role of ARs in cancer, the ligand potential of ADN in NSCLC is still superficially studied. Chung et al. illustrated the role of ADN, as a component of a polymer chain, in increasing the cellular uptake of the polymeric carrier in cancer cells and elucidated the reduction in cellular uptake of nucleic cargo when cells were pre-treated with free ADN [12]. However, they did not gain a deep understanding of the role of ARs in drug delivery. Later, Swami et al. profoundly reported improved efficacy of and-conjugated solid lipid nanoparticles in prostate and breast cancers vis-à-vis their native counterparts [7]. The results appear consistent with prior research but they need to be validated with NSCLC. Hence, it is mandatory to prove whether ADN ligand conjugated nanoparticles can assist in targeting NSCLC or not.

Poor prognosis in the early stages of cancer makes treatment of NSCLC difficult in later stages with monotherapy with platinum-based drugs. Therefore, Docetaxel (DTX) monotherapy is generally considered a standard line treatment when patients show progression after being treated with platinum-based chemotherapy [13]. DTX is a semisynthetic BCS Class IV, highly potent, water-insoluble taxol-derived broad-spectrum antineoplastic agent, with enhanced activity in malignant and cisplatin-resistant NSCLC. Clinically used DTX contains very high amounts of surfactants and alcohol that may preclude or limit their potential clinical application due to associated toxicities.

To elucidate the ligand potential of ADN in NSCLC, we designed ADN surface decorated PLGA nanoparticles encapsulating DTX. The Food and Drug Administration (FDA) and the European Medicines Agency (EMA) have authorized poly (lactic-co-glycolic acid) (PLGA) as a biodegradable and biocompatible polymer, raising the possibility of PLGA for sustained delivery systems. The polymer provides better control over release and degradation of the drug delivery system. There are many commercially available products based on PLGA such as Lupron Depot, Zoladex, etc. Their success rate proved the potential of using PLGA for the present research. Moreover, free carboxylic groups at flanking ends serve another advantage for conjugation of ADN without the need for any other excipient or linker. The literature supports many instances in which DTX was used in conjunction with PLGA nanoparticles, which dictate higher efficacy and less toxicity [14]. However, this study is the first-ever report on exploring the ADN-conjugated nanoparticles for effective management of NSCLC. We provided a proof-of-concept for systematically exploring DTX-loaded PLGA nanoparticles as a safe, improved, actively targeted therapeutic intervention for NSCLC using in vitro characterizations and in vivo evaluations.

## 2. Results and Discussion

### 2.1. Formulation and Characterization of DPLGA and ADN-DPLGA Nanoparticles

DTX encapsulation is responsible for increasing the mean particle size of the PLGA nanoparticles from 102.2 ± 3.23 nm to 138.43 ± 5.45 nm (Table 1).

**Figure 1:**
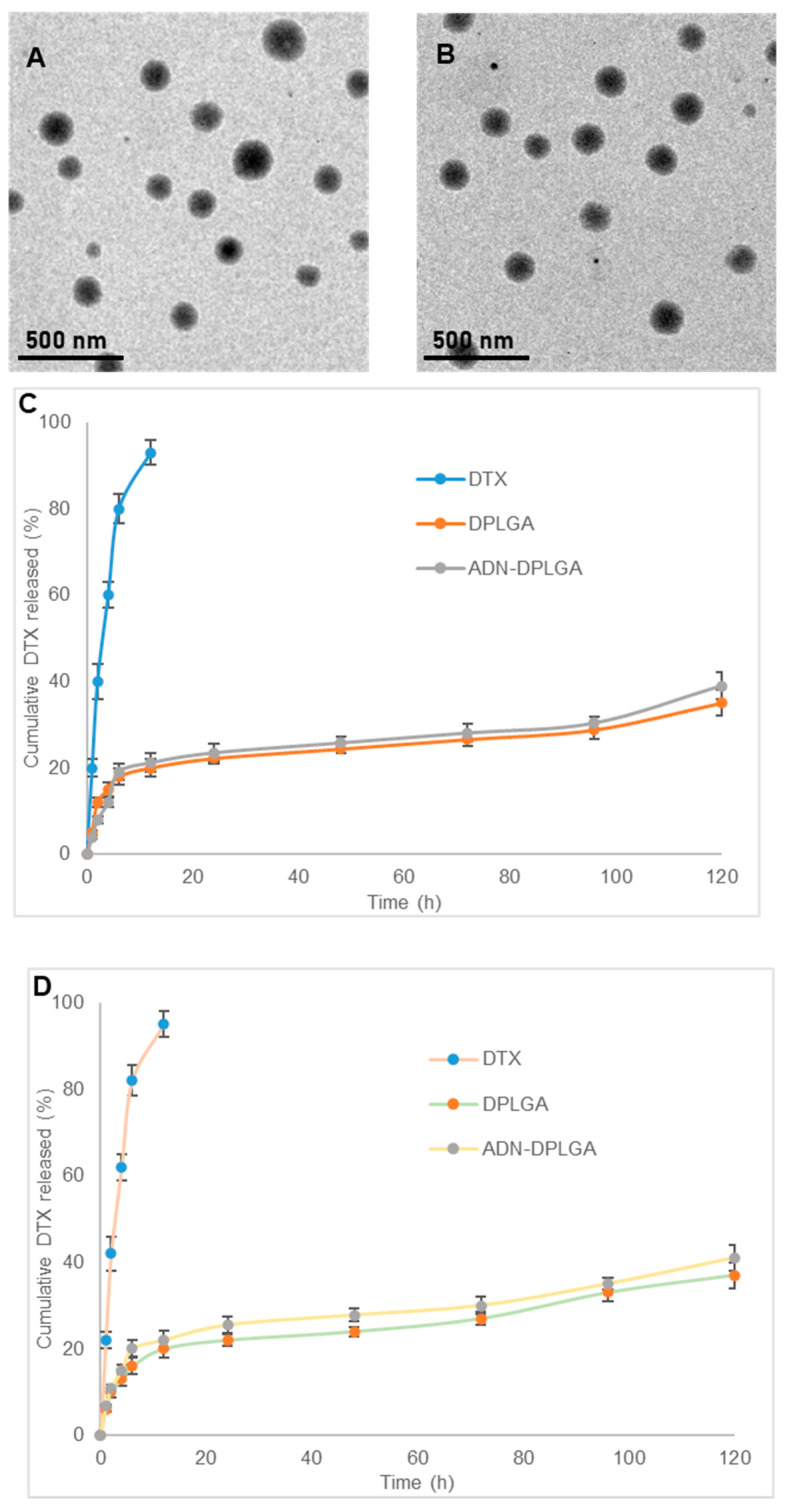
(**A**,**B**) The transmission electron microscopy (TEM) images of DPLGA and ADN-DPLGA nanoparticles, respectively. (**C**,**D**) The in vitro release profiles of DTX from pristine DTX, DPLGA, and ADN-DPLGA nanoparticles in phosphate buffer saline pH 7.4, and sodium acetate buffer (pH 5.0), respectively.

Upon conjugation, an additional increase in the mean particle size was evident owing to the attachment of multiple ADN molecules over the DPLGA nanoparticles surface (158.2 ± 6.3 nm). This is evident by the significant difference in the mean particle size of the two nanoparticles (Table 1). However, practiced peptide chemistry for the conjugation of free carboxylic groups on the surface of the DPLGA nanoparticles with the amine group of the ADN molecules resulted in decreased overall negative charge of ADN-DPLGA nanoparticles. Thus, the zeta potential of conjugated nanoparticles (ADN-DPLGA) was significantly lower (−11.7 ± 1.4) than that of non-conjugated particles (DPLGA, −16.7 ± 2.3 mV). Encapsulation efficiency expressed as % was observed as 80.12 ± 1.98 and 84.4 ± 2.61%, respectively, for DPLGA and ADN-DPLGA nanoparticles. An earlier report on multicomponent PLGA nanoparticles of docetaxel has shown entrapment efficiency of 69–75% [15]. There was no significant change in the DTX encapsulation after ADN decoration over the DPLGA nanoparticles, as illustrated in Table 1. TEM images of DPLGA Nanoparticles and ADN-DPLGA nanoparticles (Figure 1A,B) indicate that nanoparticles are spherical and have uniform size distribution. The conjugation did not affect the size and morphology of the particles.

### 2.2. Conjugation Efficiency

The extent of conjugation of amine groups of ADN with free carboxylic groups of PLGA was assessed using the colorimetry method [7]. The results were very encouraging, presenting around 75% conjugation efficiency. Higher conjugation efficiency is also the cause of the larger size of ADN-DPLGA nanoparticles observed in size measurements and TEM images shown in earlier sections.

### 2.3. In Vitro Release Studies

In vitro release of DTX from pristine DTX suspension in two different pH media, namely pH 7.4 phosphate buffer saline and pH 5.0 sodium acetate buffer, was almost complete within 12 h (>95%) (Figure 1C,D). The developed PLGA nanoparticles showed a biphasic release pattern, indicated by initial burst release followed by sustained and slow release over a prolonged period. Approximately 20 ± 2% and 21.2 ± 1.8% of DTX were released from DPLGA and ADN-DPLGA nanoparticles, respectively. At the end of day 1, only 22.5 ± 1.3 and 23.5 ± 1.2% DTX was released in phosphate buffer saline. However, in the next 4 days, only an additional 13–16% of DTX was released. The initial rapid release of DTX can be attributed to the dissolution of DTX present on the surface of the nanoparticles. Similar phenomena were seen in sodium acetate buffer as well, for both nanoparticle formulations. However, the release in sodium acetate buffer was slightly faster than the release in phosphate buffer. This may facilitate the faster release of DTX from the nanoparticles once they are taken up by the cancer cells. However, the single-point measurement on 8th day denotes around 70% release, illustrating the degradation mechanism becoming the prominent mechanism of drug release. The findings were in accordance with the previously published reports [16,17]. An earlier report on lipid-based DTX particles showed around 80% release in 10 days [18]. When the release profile of DTX from pristine DTX suspension was compared with that of nanoparticles using the *f*_2_ similarity factor, the release patterns were dissimilar as the value of *f*_2_ was less than 50 (*f*_2_ = 15 for DTX vs. DPLGA and *f*_2_ = 13.9 for DTX vs. ADN-DPLGA in phosphate buffer saline pH 7.4 and *f*_2_ = 13.6 for DTX vs. DPLGA and *f*_2_ = 14.2 for DTX vs. ADN-DPLGA). The release profiles of DTX from DPLGA and ADN-DPLGA were similar as the *f*_2_ similarity value was 87.8 and 82.9 for phosphate buffer saline pH 7.4 and sodium acetate buffer pH 5.0, respectively.

### 2.4. In Vitro Cell-Based Assays

#### 2.4.1. In Vitro MTT Assay for Calculation of IC_50_ (Half Maximal Inhibitory Concentration)

**Figure 2:**
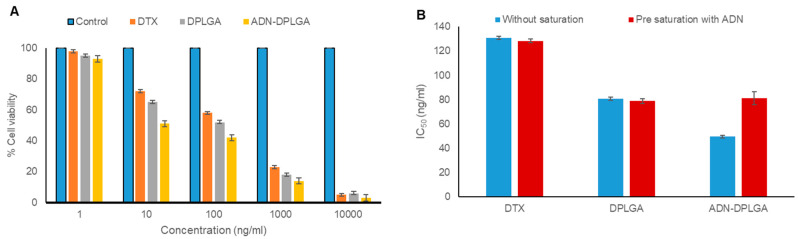
In vitro cell line studies. (**A**) Cell viability (%) of A549 cell lines treated with pristine DTX, DPLGA, and ADN-DPLGA nanoparticles. (**B**) Receptor competition assay outcomes showed an increase in the IC_50_ of ADN-DPLGA nanoparticles after A549 cells were treated with free ADN (pre-saturation), causing blockage of ADN receptors on the cells. Thus, causing a reduction in the uptake of nanoparticles by the cells results in decreased efficacy. Values represent the mean of six determinations, and error bars indicate standard deviation.

A concentration-dependent toxicity profile of the formulation was evident in the MTT assay on the A549 cell line. However, intraformational differences revealed higher activity, in terms of lower IC_50_ values, in the case of PLGA nanoparticles compared to pristine DTX treatment (Figure 2).

IC_50_ values were found to be 130.83, 80.72, and 49.50 ng/mL for pristine DTX, DPLGA, and ADN-DPLGA nanoparticles, respectively, after 48 h. The efficacy of DTX was significantly increased after being encapsulated in nanoparticles. We speculate that this might be due to the small particle size of nanoparticles resulting in higher internalization. Previous literature reported having 16-fold overexpression of ADN receptors in A549 [19]. This overexpression of the ADN receptor might be the reason for the higher retention of ADN-DPLGA nanoparticles in A549 cells due to ligand-mediated internalization. The higher efficacy in ADN-DPLGA was substantiated by the higher/rapid release of DTX in acidic pH, i.e., cancer cells (as presented in the release profile investigation in previous sections).

#### 2.4.2. Receptor Competition Assay

The role of ADN receptors in the uptake of ADN-DPLGA nanoparticles was assessed using a receptor competition assay. Results were found to be in favor of the proposed hypothesis. It is observed that IC_50_ values were notably amplified (*p* < 0.001) after saturation of ADN receptors with free ADN, as shown in Figure 2B. Free ADN exposure to A549 cells caused blockage of ARs, causing a reduction in the receptor-mediated endocytosis of the ADN-DPLGA nanoparticles. These findings support the notion that uptake of the ADN-DPLGA nanoparticles is influenced by the overexpression of ADN receptors over A549 cells that represent an example of non-small cells causing lung cancer. Previously chen et al. also documented the effect of folic acid–folic acid receptor interaction

#### 2.4.3. Cellular Uptake of Nanoparticles

**Figure 3:**
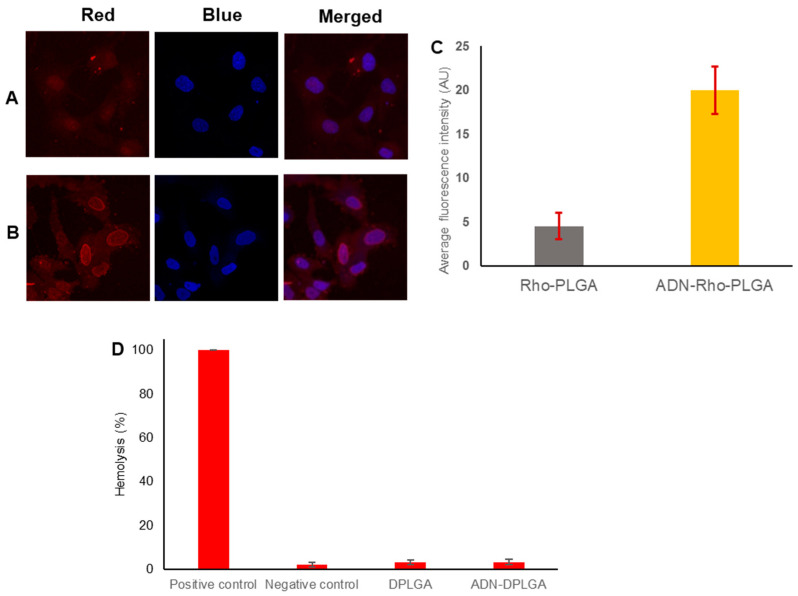
Cellular uptake and distribution of rhodamine 6G labeled DPLGA and ADN-DPLGA nanoparticles in A59 cells. (**A**,**B**) Representative CLSM images of A549 cells treated with rhodamine 6G labeled PLGA (Rho-PLGA) and ADN-RhoPLGA nanoparticles for 2 h. The cell nucleus was stained with Hoechst 33342. (**C**) Comparison of average fluorescence intensities for quantitative evaluation. The average fluorescence intensity of rhodamine was calculated using Image J software showing significantly higher uptake of ADN-PLGA nanoparticles by A549 cells. (**D**) represents the hemocompatibility analysis of DPLGA and ADN-PLGA nanoparticles when treated with red blood cells of rats. Positive control represents the treatment with Triton resulting in complete rupture of red blood cells causing maximum hemolysis. Negative control cells were treated with DMSO in phosphate buffer saline. The data represents the mean of 6 determinations, and error bars represent the standard deviation.

A549, an epithelial carcinoma cell line, is commonly used as a model to study non-small-cell lung cancer [20]. Moreover, as already discussed in the previous results there is around 16-fold overexpression of ARs over A549 cell lines [19]. Following the literature evidence, we too observed higher uptake in the case of ADN-RhoPLGA nanoparticles due to selective uptake of ADN conjugated nanoparticles through highly specific and effective receptor-mediated endocytosis (Figure 3). These findings also corroborated our previous results obtained in the MTT assay. Similar inferences were drawn in several previous publications, where the authors illustrated the receptor–ligand interaction as the crucial factor for internalization of the nanoparticles to cancer cells [21,22]. Other than ARs, there are many receptors that were highlighted in previous research, assisting in cellular uptake of nanoparticles, i.e., transferrin, lactoferrin, sigma receptors, folic acid receptors, etc. [23,24,25].

#### 2.4.4. Hemocompatibility Analysis to Estimate Biocompatibility of Nanoparticles

Outlining the interaction of developed nanoparticles with red blood cells is an essential step toward establishing the safety of the product and the plausibility of utilizing the polymeric nanoparticles as delivery tools for several other therapeutic and biomedical applications. Formulations are composed of biocompatible and biodegradable. Therefore, we expect them to be safe for the blood cells. In the present investigation, the hemolytic effects of DPLGA nanoparticles and ADN-DPLGA nanoparticles were compared with Triton in PBS (1% *w*/*v*, positive control) and DMSO in PBS (0.1% *v*/*v*, negative control) (Figure 3D). In general, hemolysis less than 10% is considered non-hemolytic and, therefore, safe and biocompatible. In the present investigation, the DPLGA nanoparticle formulation and ADN-DPLGA nanoparticles showed 3.1 and 3.2% hemolysis. Therefore, the developed polymeric nanoparticles are considered safe for systemic administration for the treatment of NSCLC [26,27].

### 2.5. In Vivo Pharmacokinetics, Biodistribution, and Acute Toxicity Testing

#### 2.5.1. Pharmacokinetic Studies

**Figure 4:**
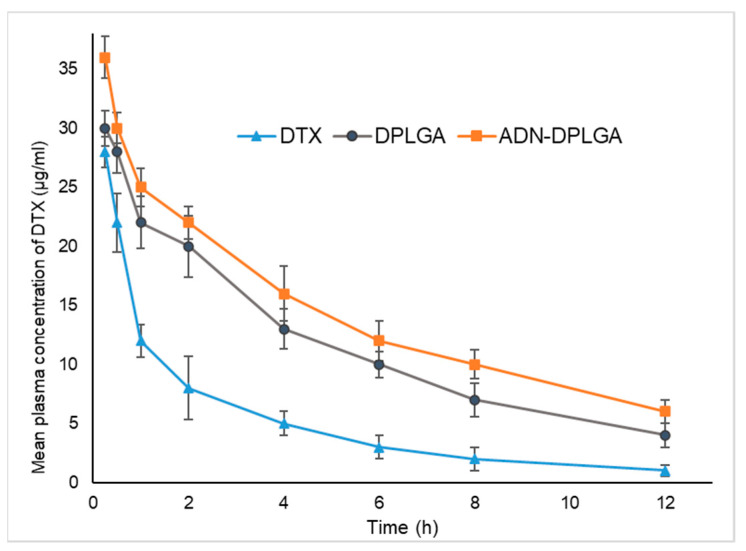
The mean plasma docetaxel concentration vs. time profile after a single intravenous injection of three different formulations, namely Docepar^®^, DPLGA, ADN-DPLGA nanoparticles equivalent to DTX (5 mg/kg). More retention and slower excretion were seen for PLGA formulations (DPLGA and ADN-DPLGA), thus, causing a larger area under curve (AUC).

The mean plasma concentration vs. time profiles after single intravenous injection of DPLGA nanoparticles, ADN-DPLGA nanoparticles, and Docepar^®^ (Parenteral Drugs India Ltd., Mumbai, India) in rats are shown in Figure 4.

It is presumed that hydrophobic surfaces tend to face early clearance from systemic circulation. Accordingly, in the present study, we observed much earlier clearance of the DTX compared to the ADN-DPLGA nanoparticles. Since ADN is a hydrophilic molecule containing sugar, it decreases RES uptake leading to reduced clearance and higher area under the curve (AUC). However, higher AUC in the case of unconjugated DPLGA nanoparticles is debatable. We speculate that it might be due to the negative charge on the surface of PLGA nanoparticles. The negative charge of the particles allows them to circumvent the RES uptake resulting in significantly prolonged systemic circulation compared to naïve drugs [28].

It can be seen that, compared with pure DTX, DTX nanoparticles, both DPLGA and ADN-DPLGA exhibited altered pharmacokinetic distribution of DTX in vivo and showed remarkably higher and prolonged plasma concentrations. The DPLGA nanoparticles exhibit almost ~3.38 times higher AUC (μg/mL·h) as compared to the pure drug ($AUC{\;}_{0}^{\infty }$_DTX_: 8.10 vs. $AUC{\;}_{0}^{\infty }$ _DPLGA_: 27.41) while ADN-DPLGA nanoparticles show ~4.51 times higher AUC as compared to the pure drug ($AUC{\;}_{0}^{\infty }$ _DTX_: 8.10 vs. $AUC{\;}_{0}^{\infty }$ _DPLGA_: 36.63). Though, the insignificant difference in mean plasma concentration was evident among the two tested PLGA formulations. The findings were in agreement with the previous literature [7,29].

#### 2.5.2. Tissue Distribution Analysis

In vivo biodistribution behavior of DTX post intravenous administration of the DTX nanoparticles (both ADN-DPLGA and DPLGA) in rats was investigated and compared with that of DTX commercially available injection as a control. The amounts of the drug distributed in the heart, liver, spleen, lung, and kidney were measured at different pre-determined time points.

**Figure 5:**
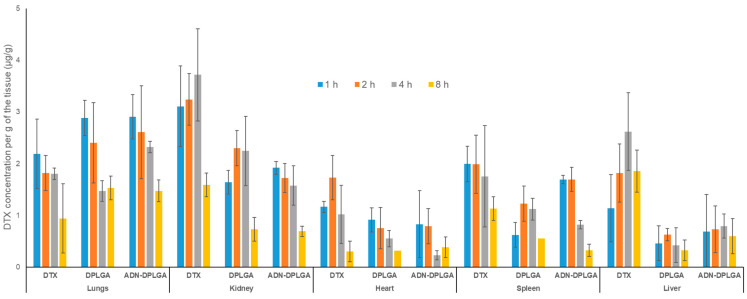
Tissue distribution studies. The concentration of DTX in various tissues after administration of three different formulations, namely, Docepar^®^, DPLGA, ADN-DPLGA nanoparticles equivalent to DTX (5 mg/kg) at four different time intervals, namely, 1, 2, 4, and 8 h. Data represents the mean of 6 determinations and error bars represent standard deviation.

New and novel nanoparticulate drug delivery systems overcome nonspecific distribution hurdles by targeting the drug to the related organ/cells. Biodistribution studies help predict the fate of nanoparticulate formulations; consequently, one can determine the exposure of different drug titers in various organs. This is an important finding in understanding the toxicity profile of the formulation. The current study findings indicate that exposure to different tissues is minimal compared to native clinical formulation, i.e., Docepar^®^. There is an insignificant difference among the PLGA formulations owing to similar basic characteristics of the formulation (Figure 5). Swami et al. explained that through biodistribution, organs are exposed to elevated levels of drug concentrations leading to toxicity. Hence correlating the drug exposure with toxicity marker gives a holistic view of the targeting to toxicity potential of a formulation.

To have a better understanding of the targeting efficiency (*Te*) of the DTX from both the nanoparticles, namely, DPLGA as well as ADN-DPLGA, parameter *Te* was calculated [30,31]. The *Te* demonstrates the ability of the delivery system to reach the target and non-target tissues. The *Te* values indicate preferential accumulation of nanoparticles in the lung tissues compared to the pristine DTX (Table 2).

These results indicate the accumulation of DTX nanoparticles in the lungs. However, there is an insignificant difference between the accumulation of ADN-DPLGA and DPLGA nanoparticles in the lungs. Though results of cell uptake studies clearly show a preferential uptake of ADN-DPLGA nanoparticles by the cancerous lung cells. ADN-DPLGA nanoparticles are the adenosine-conjugated nanoparticles that are expected to preferentially accumulate in the cancerous lung cells that exhibit over-expressed ARs. However, the present study was carried out in non-cancerous, healthy animals with lung ARs. The absence of overexpressed adenosine receptors in healthy animals seems to be the reason for equivalent tissue accumulation of the ADN-DPLGA and DPLGA nanoparticles in the lungs. However, this study revealed a key component for designing future studies on disease/cancer models to understand adjoining effects of the ADN ligand (on nanoparticle surfaces) and overexpressed ARs (on lung cancer cells) on the migration of ADN-DPLGA nanoparticles.

#### 2.5.3. In Vivo Toxicity Evaluations

**Figure 6:**
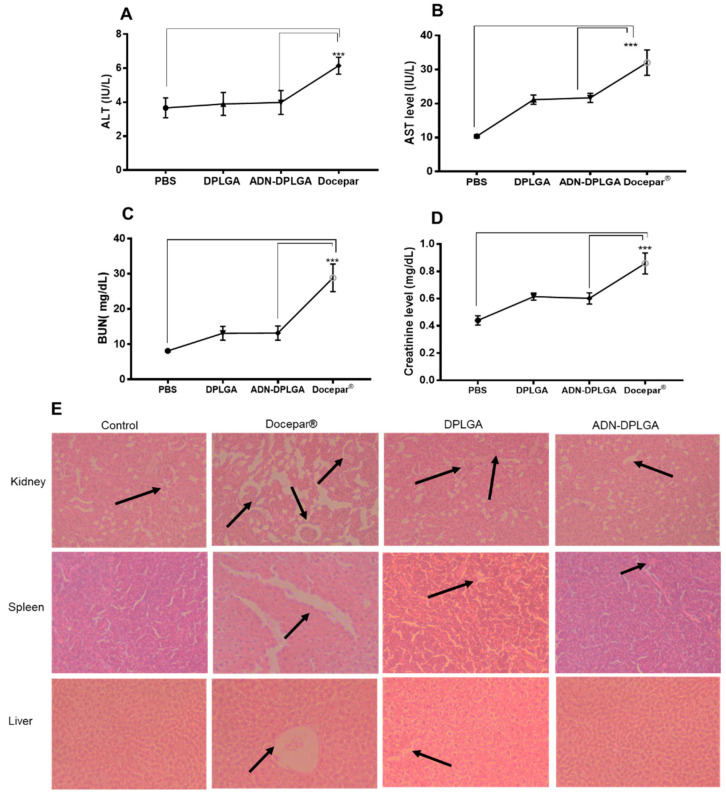
In vivo toxicity studies. (**A**–**D**) The serum biochemical markers levels representing ALT, AST, BUN, and creatinine, respectively, for four different groups of animals administered with untreated normal control (phosphate buffer saline, PBS), Docepar^®^, DPLGA, and ADN-DPLGA nanoparticles. Asterisk in biochemical studies signifies statistical limits in biochemical marker graphical representations: *** represents significant difference at *p* < 0.001, respectively, by Newman–Keuls analysis following ANOVA at 95% confidence limit. (**E**) Histological evaluations of different collected organs from the animals for toxicity investigations. Arrows indicate histological changes.

Docepar^®^, a clinically used formulation of DTX, utilizes a cocktail of surfactant and alcohol to solubilize the hydrophobic DTX to avoid drug precipitation in vitro and the systemic circulation after intravenous administration. However, the formulation is known for its toxic effects, such as hypersensitivity reactions, tissue toxicity, etc., which coincide with the native side effects of the DTX. Hence, assessing our developed formulations’ toxicities and comparing them with the clinically used formulation is of utmost necessity. Variations in serum toxicity markers to analyze the abnormality in the blood hepatobiliary system (ALT, AST) and kidney (BUN, creatinine) exemplified significant improvement (*p* < 0.05) when compared with the developed nanoparticles, as presented in Figure 6.

Docepar^®^ showed significantly higher toxicity (*p* < 0.001) as compared to PLGA formulations due to already stated reasons. Though insignificant, (*p* > 0.05) a difference between the DPLGA and ADN-DPLGA nanoparticles was evident. The histology evaluations also corroborated higher toxicity. The histological evaluations of organ (kidney, liver, and spleen) specimens revealed a normal pattern of morphology in the case of the control group, DPLGA nanoparticles, and ADN-DPLGA nanoparticles. However, prominent characteristic features were perceived in the liver (hepatocytes degeneration and infiltrations), spleen (splenocytes damage), and kidney (necrotic tubules and debris).

## 3. Materials and Methods

### 3.1. Materials

TherDose Pharma Pvt Ltd. generously provided docetaxel (DTX) and poly(d,l-lactic-co-glycolic acid) (PLGA) with a free carboxyl end group (uncapped) and an L/G molar ratio of 50:50, (Hyderabad, Andhra Pradesh, India) and Evonik (Mumbai, Maharashtra, India), respectively. ADN, Tween 80, 1-ethyl-3-(3-dimethylaminopropyl) carbodiimide hydrochloride (EDC), N-hydroxysuccinimide (NHS), Formaldehyde, Hoechst blue 33342 Rhodamine 6G, chloroform, methanol, acetone, dichloromethane, phosphotungstic acid, mannitol, dimethyl sulfoxide (DMSO), and acetonitrile were of HPLC grade (Merck, Mumbai, Maharashtra, India). The A549 cell line was obtained from the National Centre for Cell Science (NCCS, Pune, Maharashtra, India). Dulbecco’s modified Eagle’s Medium (DMEM), fetal bovine serum MTT (3-(4,5-dimethylthiazol- 2-yl)-2,5-diphenyl tetrazolium bromide), trypsin, EDTA, 2-(N-morpholino) ethanesulfonic acid (MES), Triton, and 96-well flat bottom tissue culture plates were purchased from Himedia (Mumbai, Maharashtra, India). Dialysis tubes were purchased from Spectrum (Float-A-Lyzer (G2, Spectrum, Repligen, MA, USA).

### 3.2. Preparation of DTX-Loaded PLGA Nanoparticles (DPLGA)

DTX (10 mg) was dissolved in 2 mL of acetone and dichloromethane mixture (1:1). PLGA (100 mg) was added to the DTX solution. This oil phase was emulsified for two minutes in an ice bath with an aqueous solution containing 0.25% Tween^®^ 20 using a probe sonicator (VCX 130, Sonic and Materials, Newtown, CT, USA). After emulsification, the oil-in-water emulsion was magnetically stirred for eight hours to evaporate the organic solvent [32]. The dispersion of nanoparticles was centrifuged at 15,000 rpm for 20 min at 4 °C, then washed three times with deionized water, lyophilized (5% mannitol as a freeze-drying agent), and kept at 2–8 °C. The freeze-dried nanoparticles were characterized. Similarly, blank PLGA nanoparticles were also prepared.

### 3.3. Conjugation of ADN on the Surface of DPLGA Nanoparticles

Ten milligrams of DPLGA nanoparticles were distributed in five milliliters of 0.1 M MES buffer and incubated with NHS and EDC (1:5 *w*/*w*). The dispersion was kept under gentle stirring for 2 h at room temperature, protected from light to activate free carboxylic acid groups on the PLGA nanoparticles’ surfaces. To this, 1 mg ADN was added, mixed well, and kept for further stirring for 4 h. ADN-conjugated DTX-loaded PLGA (ADN-DPLGA) nanoparticles were collected after centrifugation (Sigma Laborentrifugen GMBH, Osterode am Harz, Germany) at 15,000 rpm for 20 min and washed thrice with distilled water to remove unconjugated ADN in the supernatant. Prepared ADN–DPLGA pellets were recollected and freeze-dried (Lab Conco, Mumbai, Maharashtra, India). Freeze-dried nanoparticles were characterized further.

The conjugation efficiency of ADN to PLGA nanoparticles was quantified using phenol-sulphuric acid calorimetry assay as reported by Swami et al. [7] and expressed as a percentage of ADN bound to DPLGA nanoparticles. For cellular uptake, the nanoparticles were prepared using the same method along with rhodamine 6G as the fluorescent marker.

### 3.4. In Vitro Characterization of DPLGA and ADN-DPLGA Nanoparticles

The prepared nanoparticles, namely DPLGA and DTX-DPLGA, were characterized for several physicochemical parameters as stated below.

#### 3.4.1. Analysis of Zeta Potential, Particle Size, and Transmission Electron Microscopy (TEM)

The developed nanoparticles, namely DPLGA and ADN-PLGA, were characterized for particle size using photon cross-correlation spectroscopy. The formulation sample was put in a clear polystyrene cuvette (path length = 1 cm) after being diluted with double distilled water to ensure that the light scattering intensity remained within the instrument’s sensitivity range. Size measurements were performed using a nano-size analyzer (Nanophox, Sympatec India Pvt. Ltd., Mumbai, Maharashtra, India) at ambient temperature [33]. Zeta potential was measured on a zeta meter (Delsa Nano C, Beckman Coulter, Tokyo, Japan) [34]. For surface topography, images of nanoparticles were captured using high-resolution TEM (JEM 200, JEOL, Tokyo, Japan). The nanoparticle dispersion was put on a carbon-coated formvar grid and stained with neutralized phosphotungstic acid (1%) before being imaged under a microscope [35].

#### 3.4.2. Entrapment Efficiency (EE, %)

EE corresponds to the percentage of DTX encapsulated within or/and adsorbed onto the DPLGA and DTX-DPLGA nanoparticles. The nanoparticles suspension was centrifuged at 5000 rpm for 5 min to settle down the precipitated drug [36]. The supernatant was collected and centrifuged (Sorvall benchtop centrifuge, ThermoScientific, Mumbai, Maharashtra, India) further at 21,000 rpm for 30 min at 4 °C to settle down the nanoparticles. The concentration of DTX in the supernatant and precipitate was calculated using the previously reported and validated RP-HPLC method [7].

#### 3.4.3. In Vitro Release of DTX in Buffers

The release of DTX was studied from pristine DTX, DPLGA nanoparticles, and ADN-DPLGA nanoparticles by suspending in a Float-A-Lyzer (G2, Spectrum, Repligen, MA, USA) in two different release media, namely phosphate buffer saline pH 7.4 and sodium acetate buffer pH 5.0 containing Tween 80 to maintain sink condition and facilitate release [37]. The tests were performed at 37 °C (n = 6). The dialyzers were placed in sealed beakers with 100 mL release media and stirred on a magnetic stirrer at 100 rpm. DTX released at different time intervals was analyzed using the validated RP-HPLC method at pre-determined time intervals by withdrawing 0.5 mL of release media over 5 days. Immediately after sampling, the volume of release media was maintained at 100 mL by replacing equal amounts of release media. Release media samples were filtered through 0.22 μm PVDF filters (Millex-VV, 13 mm, Merck, Mumbai, Maharashtra, India) and analyzed after appropriate dilution with a mobile phase of the RP-HPLC method. The dissolution profiles were compared using the *f*_2_ similarity factor.

### 3.5. In Vitro Cell-Based Assays of DPLGA and ADN-DPLGA Nanoparticles

#### 3.5.1. In Vitro Cell Toxicity (MTT Assay)

A549 (adenocarcinoma human alveolar basal epithelial cells) were selected due to the availability of overexpressed ARs. Cells were obtained from National Centre for Cell Sciences (NCCS, Pune, Maharashtra, India). Cell lines were maintained as prescribed by the ATCC guidelines. For cytotoxicity evaluation, different working dilutions of DTX in sterile phosphate buffer saline were created using a 10 mg/mL stock solution in DMSO. Cytotoxicity of all the formulations and naïve drugs was determined by MTT assay based on reduction of MTT dye (yellow) by the vital mitochondrial enzymes to blue-colored formazan product. A549 cells (1 × 10^4^ cells/well) were seeded in 96-well plates and were allowed to attach overnight by incubating at 37 °C. For assessing the cytotoxicity, cells were exposed to different dilutions of DTX formulation and standard DTX and were incubated for 48 h at 37 °C in DMEM supplemented with a 10% FBS medium. After incubation, the media were aspirated, and the cells were washed twice with phosphate buffer saline (pH 7.4). The cells were processed for MTT assay [7]. The mean % of cell viability relative to untreated cells was estimated from data from multiple experiments (n = 6). The IC_50_ value was calculated using the curve fitting method.

#### 3.5.2. Receptor Competition Assay

For competitive receptor assay, A549 cells were treated with free ligand before exposing cells with optimized formulations followed by the MTT assay as described earlier in the previous section [38]. Briefly, 1 × 10^4^ cells were co-incubated with an excess of ADN. After 30 min of incubation, cells were washed twice with phosphate buffer saline (pH 7.4), followed by treatment of the cells with nanoparticle formulations and the pristine drug DTX. After incubation (48 h at 37 °C), cells were processed for MTT assay as reported earlier [21]. A comparison was completed between the IC_50_ values from the receptor competition assay and previously obtained IC_50_ values.

#### 3.5.3. Investigations from Cellular Uptake Using Fluorescent Nanoparticles

The human non-small-cell lung cancer cell line A549 was obtained from the National Centre for Cell Sciences (NCCS, Pune, Maharashtra, India). The cells were grown in DMEM medium supplemented with 10% FBS at 37 °C with 5% CO_2_ and 95% humidity. The media was replaced every 2–3 days, and the cells were detached from the culture flask using a 0.25% trypsin–0.02% EDTA solution after reaching a confluence level of 80–90%. For visualization of the cellular internalization by confocal laser scanning microscopy (CLSM, LSM 780, Carl Zeiss MicroImaging GmbH, Jena, Germany), A549 cells were seeded in a 24-well plate at a density of 1 × 10^5^ cells per well along with a coverslip, allowed to adhere and grow for 24 h. Before the experiment, the cells were washed thrice with Dulbecco’s buffer solution. Then, the cells were incubated with rhodamine 6G (Rho) loaded Rho-PLGA nanoparticles and ADN-RhoPLGA nanoparticles dispersed in the cell culture medium at 37 °C. After 2 h treatments, the cells were washed three times with cold phosphate buffer saline and treated with Hoechst blue 33342 (100 ng/mL) for 30 min. The media was removed, and cells were washed with phosphate buffer saline, fixed with 4% formaldehyde, and mounted on a coverslip. To preserve the samples, coverslips were placed on microscope slides. The CLSM apparatus was used to capture microscopy pictures. While recording the images, the microscopy gain and offset settings were kept constant throughout the study. Fluorescence in the cells was observed in CLSM with excitation wavelengths at 525 and 548 nm and emission wavelengths at 504 and 461 nm for rhodamine 6G and Hoechst blue 33342, respectively [27]. The mean fluorescence intensities were calculated from CLSM images using Image J software and plotted graphically.

#### 3.5.4. In Vitro Hemocompatibility Assay

Biocompatibility was assessed using hemolysis testing. Biocompatibility of DPLGA and ADN-DPLGA was confirmed by incubating the formulations with red blood cells. Fresh blood was obtained from rats and centrifuged at 2500 rpm for 10 min at 4 °C in heparinized tubes. The pellet obtained after centrifugation was washed thrice with phosphate buffer saline, and cells were finally resuspended in phosphate buffer saline. In a 96-well plate, an equal volume of 100 μL of erythrocyte suspension and the nanoparticles’ dispersion were combined. The plate was incubated at 37 °C for 1 h. After 1 h, the plate was centrifuged, and the supernatant was transferred to another 96-well plate. The absorbance was measured at 540 nm using a microplate reader (Erba LisaScan EM, Transasia, Mumbai, India). The supernatant generated from the centrifuged blood sample was used as a blank, and the supernatant derived from the blood sample treated with 1% Triton *w*/*v* was utilized as a positive control. Cells treated with 0.1% *v*/*v* DMSO in phosphate buffer saline were considered a negative control. All measurements were repeated (n = 6), and the percent hemolysis was calculated [26,27].

### 3.6. In Vivo Pharmacokinetics, Biodistribution, and Acute Toxicity Studies

The experimental protocol was approved by the Institutional Animals Ethics Committee (Vidya Siri College of Pharmacy, Bangalore, Karnataka, India). The experiment was carried out in accordance with the rules for experimental animal care established by the Committee for the Purpose of Control and Supervision on Experiments on Animals (CPCSEA). The protocol approval number is VSCP/EC/1405/2021/2, with a date of approval 14 May 2021. Female Sprague Dawley rats (150–200 g) were used for pharmacokinetic and biodistribution evaluations. Female Swiss albino mice (20–25 g) were utilized for toxicity evaluation. The animals were housed in normal wire mesh plastic cages in a room kept at 22 ± 0.5 °C with a 12 h light and 12 h dark cycle, and they were fed a standard pellet diet and provided water ad libitum. Experiments were carried out between 09:00 and 17:00 h.

#### 3.6.1. Pharmacokinetic Studies

The rats were assigned to one of three treatment groups: Docepar^®^ (commercially available product), DPLGA, or ADN-DPLGA nanoparticles. Each group had six animals (n = 6). The dose equivalent to 6 mg/kg of DTX was administered intravenously (IV) [22]. At different time intervals, blood samples were withdrawn and centrifuged at a fixed speed of 10,000 rpm for 5 min at 4 °C. The plasma samples were kept at −80 °C until they were processed. DTX concentrations were measured using the previously indicated verified and calibrated HPLC technique. Mean plasma concentration vs. time profile was represented graphically, and the area under the curve (AUC) was calculated [39] for comparison purposes.

#### 3.6.2. Tissue Distribution Analysis

Animals were randomly divided into three treatment groups, namely, Docepar^®^ (A commercially available product), DPLGA, and ADN-DPLGA nanoparticles. Individual groups had an equal number of animals. Each group received a single fixed dose of respective formulations equivalent to 5 mg/kg of DTX by IV route of administration. Mice (n = 6) were sacrificed at 1, 2, 4, and 8 h of post-dose and were dissected to isolate the heart, liver, spleen, kidney, and lungs. Tissues were weighed, homogenized in phosphate buffer saline, and were stored at −80 °C until further processing [40]. DTX concentration in each tissue was assessed after extracting in the organic phase and analyzing by validated RP-HPLC as described earlier. The lung targeting ability of the DTX nanoparticles was calculated using plasma concentration data. The tissue targeting ability of the delivery system was measured based on the drug targeting efficiency (*T_e_*) calculated using the equation below.

(1) $$T_{e}=\frac{\left(AUC{\;}_{0}^{\infty }\right)Target\;tissue}{\left(AUC{\;}_{0}^{\infty }\right)\;Non\;target\;tissue}$$

#### 3.6.3. In Vivo Toxicity—Biochemical Analysis and Histopathology

To estimate drug-induced toxicities, mice were randomly divided into four different formulation groups, namely, untreated normal control (phosphate buffer saline, PBS), Docepar^®^, DPLGA, and ADN-DPLGA nanoparticles containing nanoparticles in an equal number of animals (n = 6). Formulations with a dose equivalent to 5 mg/kg of DTX were administered intravenously. The untreated normal group similarly received only normal saline [41]. Animals were humanly sacrificed after 7 days, followed by the collection of blood samples using cardiac puncture. Serum was separated, and several biochemical markers such as blood urea nitrogen (BUN), aspartate aminotransferase (AST), alanine aminotransferase (ALT), and creatinine levels were analyzed according to the instructions of the commercial kits (Sigma Aldrich, Bangalore, Karnataka, India). The vital organs, namely the liver, spleen, and kidney, were preserved in 10% formaldehyde in phosphate buffer saline, embedded in paraffin wax, and sliced into layers using a microtome (Leica, Wetzlar, Germany). The sections were observed in a light microscope (Olympus, Tokyo, Japan) after staining with Hematoxylin and Eosin (H&E).

### 3.7. Statistical Analysis

The statistical significance of the data was determined using a one-way analysis of variance (ANOVA) at a 95% confidence level. The Newman–Keul’s test for statistical significance at the 95% confidence level was used to examine any significant differences between groups.

## 4. Conclusions

Lung cancer continues to be the most lethal form of cancer today. The present investigation is a proof-of-concept for developing targeted nanoparticulate interventions for non-small lung cancer (NSCLC) overexpressing adenosine (ADN) receptors (ARs). In the current investigation, an ADN ligand having a high affinity for ARs was postulated to develop ADN-conjugated PLGA nanoparticulate formulations containing docetaxel (DTX) as a chemotherapeutic agent. A series of investigations were conducted, and inferences were drawn in favor of the developed formulation. Planned comparisons between conventional clinically used formulations, i.e., Docepar^®^ and tested formulations established the supremacy of the developed formulation over Docepar^®^.

Ligand-conjugated nanoparticulate systems offer a flexible and versatile technology that can be adapted to various drugs by modulating the process parameters to achieve the desired therapeutic response. When administered systemically, such ADN-conjugated nanoparticles can also serve as a platform technology for the active targeting of drugs to the cells with overexpressed ADN receptors with minimal non-target side effects.

## Figures and Tables

**Table 1:** Mean particle size, surface potential, and entrapment efficiency among different formulations *.

Nanoparticle Formulation	Particle Size (nm)	Zeta Potential (mV)	Entrapment Efficiency (EE, %)
PLGA	102.2 ± 3.2	−17.0 ± 3.5	NA
DPLGA	138.4 ± 5.4	−16.7 ± 2.3	80.12 ± 1.98
ADN-DPLGA	158.2 ± 6.3	−11.7 ± 1.4	79.84 ± 2.66

* Data represent the mean of six determinations ± SD.

**Table 2:** Comparative drug targeting efficiency of DTX form Docepar^®^, DPLGA, and ADN-DPLGA nanoparticles.

Organs	*T_e_* DTX	*T_e_* DPLGA	*T_e_* ADN-DPLGA
Lung	0.24	3.23	3.87
Liver	2.35	0.25	0.18
Spleen	1.23	0.32	0.36
Kidney	2.08	0.92	0.98
Heart	1.06	0.65	0.54

## Data Availability

Data is contained within the article.
